# Spatial niche formation but not malignant progression is a driving force for intratumoural heterogeneity

**DOI:** 10.1038/ncomms11845

**Published:** 2016-06-13

**Authors:** Rouven Hoefflin, Bernd Lahrmann, Gregor Warsow, Daniel Hübschmann, Cathleen Spath, Britta Walter, Xin Chen, Luisa Hofer, Stephan Macher-Goeppinger, Yanis Tolstov, Nina Korzeniewski, Anette Duensing, Carsten Grüllich, Dirk Jäger, Sven Perner, Gita Schönberg, Joanne Nyarangi-Dix, Sanjay Isaac, Gencay Hatiboglu, Dogu Teber, Boris Hadaschik, Sascha Pahernik, Wilfried Roth, Roland Eils, Matthias Schlesner, Holger Sültmann, Markus Hohenfellner, Niels Grabe, Stefan Duensing

**Affiliations:** 1 Section of Molecular Urooncology, Department of Urology, University of Heidelberg School of Medicine, Medical Faculty Heidelberg, Im Neuenheimer Feld 517, D-69120 Heidelberg, Germany; 2 Hamamatsu Tissue Imaging and Analysis (TIGA) Center, BioQuant, University of Heidelberg, Im Neuenheimer Feld 267, D-60120 Heidelberg, Germany; 3 Division of Theoretical Bioinformatics (B080), German Cancer Research Center (DKFZ), Im Neuenheimer Feld 280, D-69120 Heidelberg, Germany; 4 Department for Bioinformatics and Functional Genomics, Institute for Pharmacy and Molecular Biotechnology (IPMB) and BioQuant, University of Heidelberg, Im Neuenheimer Feld 267, D-69120 Heidelberg, Germany; 5 Department of Pediatric Immunology, Hematology and Oncology, University Hospital Heidelberg, Im Neuenheimer Feld 430, D-69120 Heidelberg, Germany; 6 National Center for Tumor Diseases, Department of Medical Oncology, Im Neuenheimer Feld 460, D-69120 Heidelberg, Germany; 7 Department of Pathology, University of Heidelberg School of Medicine, Im Neuenheimer Feld 224, D-69120 Heidelberg, Germany; 8 Department of Urology, University of Heidelberg School of Medicine, Im Neuenheimer Feld 110, D-69120 Heidelberg, Germany; 9 Center for Kidney Tumors, National Center for Tumor Diseases and University of Heidelberg School of Medicine, Im Neuenheimer Feld 460, D-69120 Heidelberg, Germany; 10 University of Pittsburgh Cancer Institute, Cancer Therapeutics Program, 5117 Centre Avenue, Pittsburgh, Pennsylvania 15232, USA; 11 Institute of Pathology, University Hospital Lübeck and Leibniz Research Center Borstel, Ratzeburger Allee 160, D-23538 Lübeck, Germany; 12 National Center for Tumor Diseases, German Cancer Research Center, Division of Cancer Genome Research, German Cancer Consortium (DKTK), Im Neuenheimer Feld 460, D-69120 Heidelberg, Germany

## Abstract

Intratumoural heterogeneity (ITH) is a major cause of cancer-associated lethality. Extensive genomic ITH has previously been reported in clear cell renal cell carcinoma (ccRCC). Here we address the question whether ITH increases with malignant progression and can hence be exploited as a prognostic marker. Unexpectedly, precision quantitative image analysis reveals that the degree of functional ITH is virtually identical between primary ccRCCs of the lowest stage and advanced, metastatic tumours. Functional ITH was found to show a stage-independent topological pattern with peak proliferative and signalling activities almost exclusively in the tumour periphery. Exome sequencing of matching peripheral and central primary tumour specimens reveals various region-specific mutations. However, these mutations cannot directly explain the zonal pattern suggesting a role of microenvironmental factors in shaping functional ITH. In conclusion, our results indicate that ITH is an early and general characteristic of malignant growth rather than a consequence of malignant progression.

## Abstract (web summary)

It has been increasingly recognised that tumours are not made up of a homogeneous population of cells. Here, the authors show heterogeneous expression of five protein markers in renal cell cancer and demonstrate that the progression of the tumour does not influence the degree of heterogeneity in the tumour.

## Graphical abstract

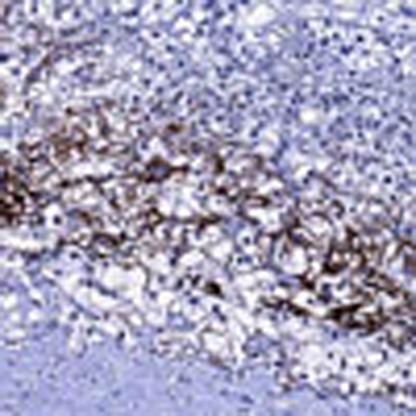

Intratumoural heterogeneity (ITH) is a result of genomic instability and causes clonal variability that drives malignant progression, therapy resistance and fatal disease outcome^1^,^2^,^3^. Elucidating the causes and consequences of ITH is therefore of utmost importance not only for cancer diagnosis and prognosis but also for new therapeutic strategies and personalized cancer patient management^4^.

Multiregion exome sequencing studies of clear cell renal cell carcinoma (ccRCC) have recently underscored the extensive genomic ITH and branched clonal evolution of this tumour type^5^,^6^. In an initial study involving a widely metastatic ccRCC, approximately 31% of the somatic mutations detected were ubiquitous. Approximately 23% were shared between sampling sites of the primary tumour whereas approximately 21% were shared between metastatic lesions, or found exclusively in one of the lesions analysed (‘private mutations'; approximately 25%)^5^. Inactivation of the *VHL* tumour suppressor gene was ubiquitously detected as expected for a truncal driver alteration. In a follow-up study^6^, the high frequency of heterogeneous mutations with most driver mutations arising in spatially separated subclones was confirmed. In addition, multiple subclones in a given tumour region were detected.

These findings raise the possibility that the evolution of tumour subclones leads to enhanced genomic ITH as a tumour progresses and that this is reflected by increased functional ITH. In this scenario, functional ITH as measured by changes in protein expression or protein phosphorylation could be a prognostic marker.

To test this hypothesis, we used a precision quantitative imaging approach as well as regional whole-exome sequencing to characterize ITH in primary ccRCCs of various stages. We did not detect any significant differences in the extent of functional and genomic ITH between ccRCC of the lowest stage in comparison to advanced tumours. However, we unexpectedly found marked and consistent spatial differences in functional ITH between the tumour centre and the tumour periphery with highest proliferation and signalling activities almost exclusively in the tumour periphery. Using whole-exome sequencing, we discovered various region-specific mutations, thus underscoring that genomic ITH also follows a spatial pattern. However, the periphery-specific mutations could not directly explain the functional ITH, which underscores the role of microenvironmental factors in Intratumoural niche formation. Collectively, our results suggest that ITH is an early and general characteristic of primary ccRCCs that does not increase with malignant progression. They support a model in which Intratumoural niches, most notably the tumour peripheral zone and the tumour centre, populated by tumour subclones with unique functional properties and mutations, are drivers of ITH.

## Results

### Phenotypic and functional ITH in ccRCC is stage-independent

**Figure 1:**
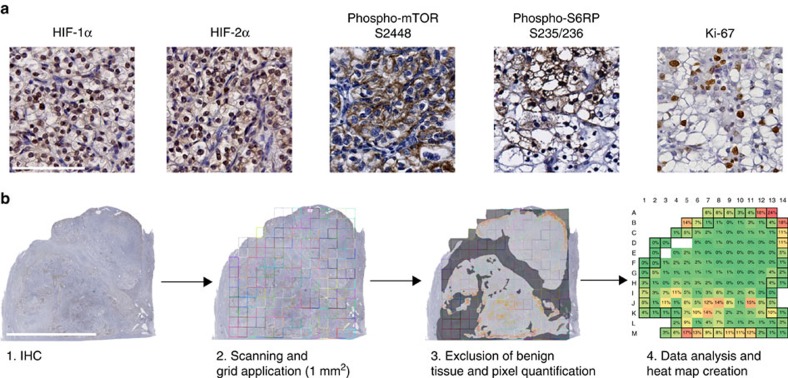
Precision quantitative image analysis workflow for the characterization of functional ITH. (**a**) Representative areas with positive immunohistochemical (IHC) staining for HIF-1α, HIF-2α, phospho-mTOR S2448, phospho-S6RP S235/236 and Ki-67. Only nuclear HIF-1α and HIF-2α expression was included in the analysis. Scale bar, 100 μm. (**b**) Overview of the quantitative IHC analysis process using a representative image of a whole-slide scanned pT1M1 tumour after phospho-mTOR S2448 staining. The image was overlaid with a virtual grid consisting of 1 mm^2^ squares that defines the tumour regions to be analysed. Non-cancerous tissue was subtracted manually (dark grey) and a positive pixel count was performed on the corrected area. Positivity per square was expressed as percentage of positive pixels. If a square contained non-tumorous tissue, the positivity was normalized to the corrected square area. Computed values were depicted as a heat map. Coloured squares represent tumour, whereas open squares indicate completely non-tumorous areas. Squares defined as peripheral tumour zone are marked by a black line. Scale bar, 1 cm.

For a quantitative image analysis of phenotypic and functional ITH, four immunohistochemical markers were used as surrogates for genomic alterations in key signalling pathways in ccRCC. These included HIF-1α and HIF-2α (*VHL* inactivation), phospho-mTOR S2448 as well as phospho-S6RP S235/236 (activating mutations in the PI3K/AKT/mTOR pathway)^5^. In addition, Ki-67 was included to assess the cellular proliferation (Fig. 1a). Primary tumours from 30 patients were analysed (Supplementary Table 1), and staining results were quantified as outlined in Fig. 1b. To detect changes in the extent of functional ITH associated with malignant progression, we analysed a spectrum of tumours ranging from small pT1 (*n*=9) tumours to advanced pT3/4 tumours (*n*=11) with or without lymphatic or distant metastasis. A very rare subgroup of small T1 tumours with synchronous distant metastasis (*n*=10) was included with the idea that these tumours would contain features of more advanced ccRCCs. Since our intention was to compare small renal tumours with widely advanced tumours, pT2 RCCs were deliberately excluded.

**Figure 2:**
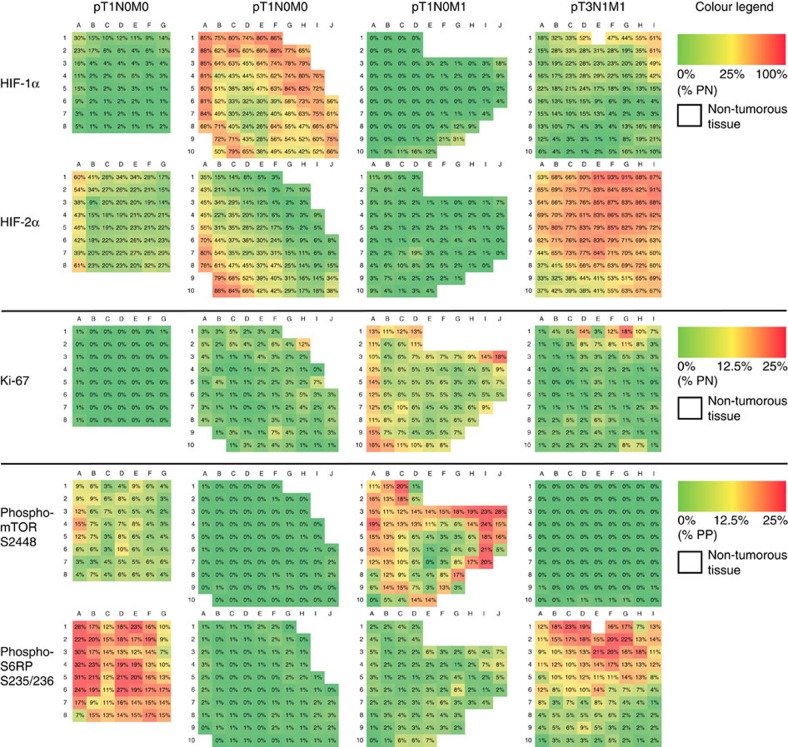
Functional ITH is independent from tumour stage. Representative heat maps of biomarker expression (HIF-1α, HIF-2α, phospho-mTOR S2448, phospho-S6RP S235/236 and Ki-67) in different prognostic subgroups. Positivity per square was calculated using the positive nuclear count for HIF-1α, HIF-2α and Ki-67, and expressed as percentage positive nuclei (% PN). Colours correspond to % PN and range from absent (0% PN; green) to high (100% PN; red) for HIF-1α and HIF-2α. Ki-67 showed a maximum of 25% PN and thus is represented by a smaller range from 0% PN (green) to 25% PN (red). Cytoplasmic staining for phospho-mTOR S2448 and phospho-S6RP S235/236 was analysed as shown in Fig. 1 and expressed as percentage positive pixels (% PP). Note that a square with 25% PP is considered completely positive. White squares represent completely non-cancerous tissue and were excluded from analysis.

A total of 9,315 data points were collected from five directly adjacent histological sections from each of the 30 tumours after two-dimensional alignment to allow a direct comparison of the same tumour area. An average of 75 virtual 1 mm^2^ squares per stained section were analysed using either a positive nuclear count (PNC) algorithm (HIF-1α, HIF-2α and Ki-67) or a positive pixel count (PPC) algorithm (phospho-mTOR S2448 and phospho-S6RP S235/236). The latter algorithm was designed to measure every pixel per 1 mm^2^ square based on its spectral characteristics (brown for positive staining versus other colours for negative staining). Non-cancerous regions were excluded from quantification. Staining results for each square were expressed as either percentage positive nuclei or percentage positive pixels. If a 1 mm^2^ square contained non-cancerous tissue, the percentage of positive pixels was normalized to the reduced area. Staining results were expressed as percentage positivity per square and represented as heat maps with negative staining in green and the highest positivity in red (Fig. 2; note that the highest positivity reached differed between PNC and PPC). Unexpectedly, a first comparison of heat maps obtained from tumours of various stages did not indicate any major differences in functional ITH between tumour subgroups (Fig. 2).

**Figure 3:**
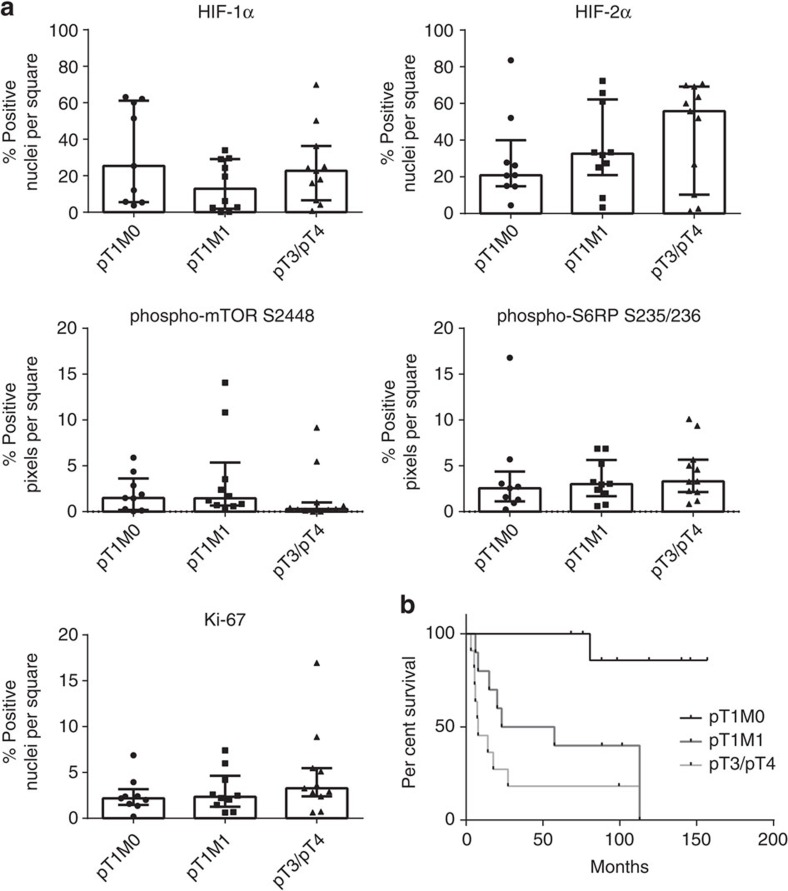
Quantification of functional ITH in prognostic ccRCC subgroups. (**a**) Scatter plots showing median±interquartile range of the mean percentage positive pixels (phospho-mTOR S2448 and phospho-S6RP S235/236) or mean percentage positive nuclei (HIF-1α, HIF-2α and Ki-67) per tumour. No statistically significant differences were observed between subgroups (*P*>0.05, Kruskal–Wallis test for multiple comparisons). (**b**) Kaplan–Meier cancer-specific survival analysis of the patients.

To corroborate this notion, we first analysed expression of individual markers for each prognostic subgroup (Fig. 3a). No statistically significant differences were detected when mean percentages of positive nuclei or pixels per square in pT1M0 tumours were compared with pT1M1 or pT3/T4 tumours (*P*>0.05, Kruskal–Wallis test for multiple comparisons). This result was not due to patient selection, since the cancer-specific survival analysis confirmed the differences in patient prognosis (Fig. 3b).

**Figure 4:**
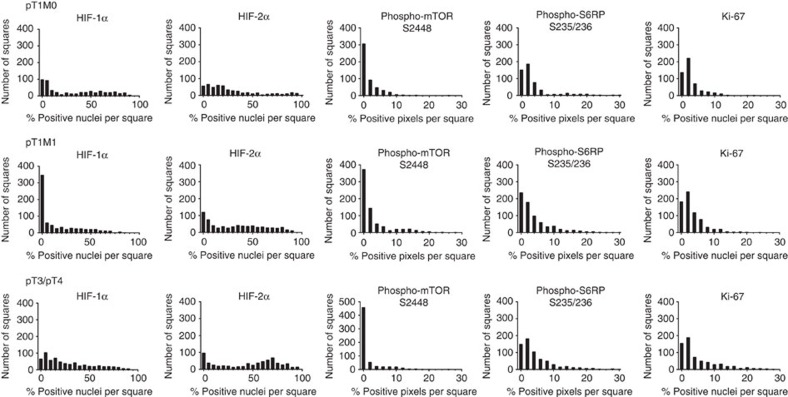
Heterogeneous expression of markers of functional ITH in prognostic ccRCC subgroups. Cumulative histograms of tumour subgroups representing the number of squares that contained a certain percentage of positive pixels or positive nuclei are shown. None of the marker distribution passed the D'Agostino–Pearson omnibus normality test indicating a non-Gaussian distribution and therefore a heterogeneous expression pattern in all three prognostic subgroups.

Next, we displayed the distribution of the five tissue markers as histogram plots (Fig. 4). These histograms represent the number of 1 mm^2^ squares that contained a certain percentage of positive pixels or positive nuclei. For an analysis of individual marker distribution, we used the D'Agostino–Pearson omnibus test. This test measures goodness-of-fit and determines how symmetrical the distribution of the data is and whether the data distribution follows a Gaussian distribution. A homogeneous marker distribution throughout an entire tumour sample would be reflected in a histogram with a normal, or Gaussian distribution, and the test would be passed. The D'Agostino–Pearson test does not only provide information about whether a distribution follows a Gaussian distribution or not but also calculates *P* values to assess the probability that a negative or positive test result is correct. In total, 72% of the samples were significantly D'Agostino–Pearson negative (*P*<0.05; D'Agostino–Pearson test) and were hence considered to be heterogeneous. Twenty-eight percentage of the samples passed the test; however, not with a significant *P* value (*P*>0.05; D'Agostino–Pearson test; Supplementary Table 2).

We next sought to directly quantify the degree of functional ITH in the various prognostic subgroups. To this end, we used two quantitative scores, the s.d. of the PNCs and PPCs from all squares of a given tumour sample stained for a specific marker and the maximum-mean (MAX-μ) score. The latter is particularly useful to identify outlier regions of positive marker staining within the tumour area analysed. In addition, we used two established biodiversity indices, the Shannon and the inverse Simpson index, to characterize functional ITH.

**Figure 5:**
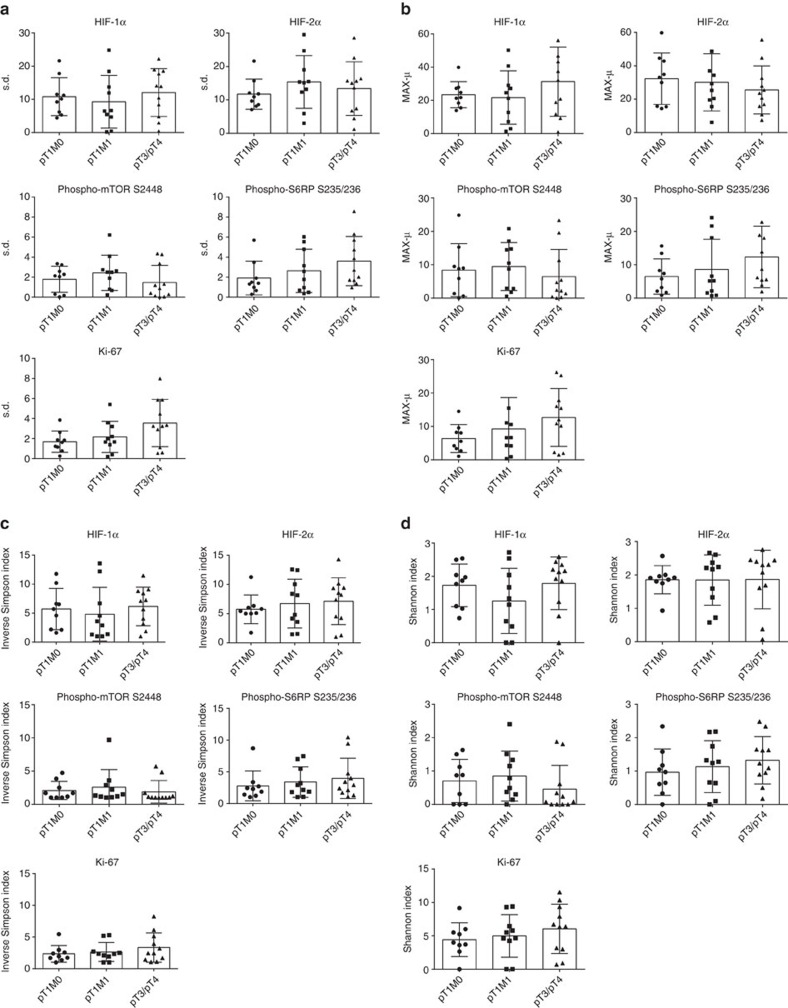
Functional ITH is stage independend as measured by heterogeneity scores and biodiversity indices. (**a**–**d**) Scatter plots representing mean±s.d. and individual values of the heterogeneity scores s.d. (**a**), MAX-μ (**b**), inverse Simpson index (**c**) and Shannon index (**d**) for the indicated biomarkers and prognostic subgroups.

S.d. and MAX-μ scores as well as Shannon and inverse Simpson indices were calculated for all five markers for each of the 30 tumour specimens (Fig. 5). No statistically significant differences between different prognostic subgroups were detected (*P*>0.05, Kruskal–Wallis test for multiple comparisons). In addition, we were not able to corroborate a statistically significant correlation between ITH scores and histological tumour grade (*P*>0.05; Mann–Whitney *U*-test; Supplementary Table 3).

In summary, our data indicate that functional ITH exists already in primary ccRCCs of the lowest stage and does not increase with malignant progression.

### ITH is unaffected by changes in signalling cascades

We next performed a correlation analysis of all markers and tumours using directly corresponding virtual squares of the adjacent tissue sections (Supplementary Table 4). In both pT1M0 and pT1M1 tumours, a moderate-to-good positive correlation (*r*=0.4–0.7; Spearman's rank correlation coefficient) for HIF-1α and HIF-2α as well as co-expression of phospho-mTOR S2448 and phospho-S6RP S235/236 was found. In addition, there was a negative correlation (*r*=−0.51; Spearman's rank correlation coefficient) for HIF-1α and phospho-mTOR S2448 expression in pT1M0 tumours. A negative regulation of mTORC1 by HIF-1α has previously been reported through the HIF-1α transcriptional target REDD1 (ref. 7), and it remains to be determined if this is the underlying mechanism for this observation. Tumours of pT1M1 stage showed a positive correlation between HIF-1α and phospho-S6RP S235/236 expression (*r*=0.51; Spearman's rank correlation coefficient). Remarkably, however, none of these correlations was found in pT3/4 ccRCCs. Instead, there was a negative correlation between HIF-2α expression and the proliferation marker Ki-67 (*r*=−0.46; Spearman's rank correlation coefficient)^8^. No significant correlations were detected when all tumours combined were analysed.

Taken together, these results indicate that apparent changes in tissue marker correlations associated with malignant progression (highlighted in Supplementary Fig. 1) are not reflected by changes in functional ITH as determined by our quantitative image analysis.

### Tumour periphery and centre are distinct topological niches

**Figure 6:**
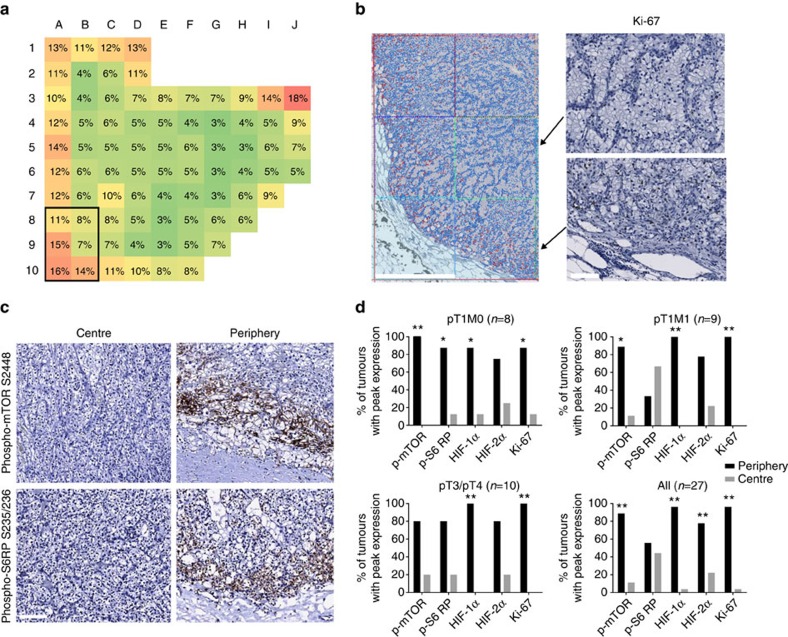
Topological differences in functional ITH define tumour centre and periphery as distinct spatial niches. (**a**,**b**) Heat map and immunohistochemical staining of a representative ccRCC for Ki-67. A representative picture showing the PNC analysis is shown in (**b**, left panel). Positive nuclei are false coloured in red, negative nuclei blue. Scale bar, 1 mm. High-power views of squares B9 and B10 immunostained for Ki-67 are shown (**b**, right panels). Note the differences in proliferating cells between squares B9 (centre) and B10 (periphery). Scale bar, 100 μm. (**c**) Representative immunohistochemical stainings for phospho-mTOR S2448 and phospho-S6RP S235/236 in the tumour centre and the tumour periphery. Scale bar, 100 μm. (**d**) Each bar shows the percentage of tumours with peak marker expression in the central (grey bars) versus peripheral (black bars) tumour zone. Significance was assessed using the Bernoulli trial (*P<0.05, **P<0.01).

When we analysed the expression of Ki-67 as a marker of cellular proliferation, we noticed that proliferating cells were preferentially found in the tumour periphery (Fig. 6a,b). Markers indicating an activated PI3K/mTOR pathway were found to follow this pattern with highest expression in the periphery of the tumours (Fig. 6c). We therefore systematically analysed all tumours and defined the peripheral zone as the outermost squares of each tumour (Fig. 1) and determined the localization of the square with the peak expression for all markers used (Fig. 6d). A total of 4,475 peripheral zone and 4,425 central zone squares were evaluated. We found that in the vast majority of tumours, the peak marker expression was in the peripheral tumour zone (*P*<0.05; Bernoulli trial). The only exception was the expression of phospho-S6RP S235/236 in pT1M1 tumours, where the majority of tumours showed a peak expression in the central zone. This result and the fact that tumours were not uniformly negative in the centre (Fig. 2) rule out that non-specific effects of tissue processing are responsible for our findings. When we compared pT1M0 with pT1M1 and pT3/4 tumours, we did not find any significant differences between the prognostic subgroups with respect to the peripheral and central zone expression of markers (*P*>0.05, Fisher exact test, two-tailed). Next, we tested whether an unequal admixture of non-tumorous cells may account for the topological differences that were detected. Centre and peripheral zones of 24 tumours were stained for CD31 to measure microvessel density and CD45 to measure leukocyte infiltration (Supplementary Fig. 2). No significant differences between the tumour centre and periphery were detected (*P*>0.05; Mann–Whitney *U*-test), thus underscoring that spatial ITH was not due to the admixture of non-tumorous cells or differences in intratumoural microvessel density (see also analysis of mutant allele frequencies below).

These results indicate that the tumour periphery and centre represent distinct topological niches that differ functionally and that are retained during malignant progression.

### Tumour periphery and centre harbour distinct somatic mutations

**Figure 7:**
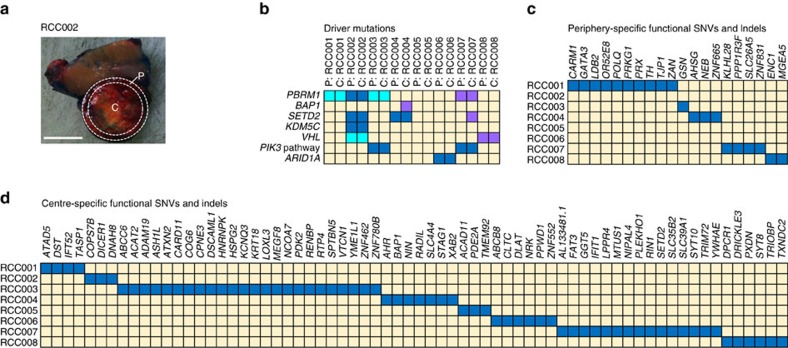
Whole-exome sequencing reveals distinct mutations in the tumour centre and periphery. (**a**) Macroscopic view of RCC002 with centre (C) and periphery (P) labelled as dissected. Scale bar, 1 cm. (**b**) Map of driver mutations found in the tumour periphery or tumour centre of all samples. Colour indicates the presence of a mutation (blue, nonsynonymous SNV; lavender, frameshift; cyan, stop gain/loss). Yellow indicates the absence of a mutation. (**c**,**d**) Maps of functional SNVs and indels (blue) specific for the tumour periphery (**c**) or centre (**d**).

We next sought to corroborate whether the differences between the tumour periphery and the tumour centre with respect to functional ITH are based on genetic alterations and/or distinct mutational processes. To this end, we performed whole-exome sequencing of eight consecutive ccRCCs with different pathological stages ranging from pT1 to pT4 (Supplementary Table 5). The zonal pattern of functional heterogeneity was maintained in all tumours (Supplementary Fig. 3). Tumour periphery and centre were macrodissected (Fig. 7a), and whole exomes were sequenced with an average coverage of 50.3 × . We first analysed known truncal driver mutations and recurrently mutated genes and found a total of 28 mutations in seven known ccRCC driver genes and pathways (Fig. 7b)^9^,^10^,^11^,^12^. Driver mutations were typically found in both the peripheral and central tumour areas.

For every patient, single-nucleotide variants (SNVs) detected only in the respective peripheral zone sample were designated as peripheral and SNVs detected only in the respective central zone sample were designated as central. Variants that occurred recurrently in at least six of the eight patients or that were dbSNP-associated without clinical context were removed. SNVs were called functional, when they were nonsynonymous, occurred within splice sites or lead to gain or loss of stop codons.

For an analysis of mutational signatures, SNVs were grouped into 96 different categories according to their trinucleotide context and the type of base exchange as previously described^13^.

We detected a total of 88 functional SNVs and indels that were specific for either the peripheral zone or the central zone of the tumours analysed (Fig. 7c,d). There was no correlation between the number of centre- or periphery-specific mutations and tumour stage or grade (Supplementary Table 5).

Whereas the central zone specimens from eight ccRCCs contained on average 8.5±2.6 (mean±s.e.m.) specific functional SNVs and indels, the eight peripheral zone specimens contained on average 2.5±1.2 (mean±s.e.m.) specific functional SNVs. There were no recurrently mutated genes in the tumour peripheral zone that could directly explain the consistently higher proliferation rate and signalling activities detected there. Instead, mutations were found, for example, in genes involved in chromatin remodelling (*CARM1* and *GATA3*), DNA replication and repair (*POLQ*), and cell migration or cytoskeletal organization (*TJP1*, *GSN* and *ENC1*). These results suggest that, despite the presence of unique mutations in the tumour periphery in six of eight tumours, the enhanced cellular proliferation and signalling activity represent mainly functional events. An analysis of the mutant allele frequencies between the centre and periphery showed no major differences, thus further supporting the notion that our findings were not due to unequal admixture of non-tumorous cells (Supplementary Fig. 4).

**Figure 8:**
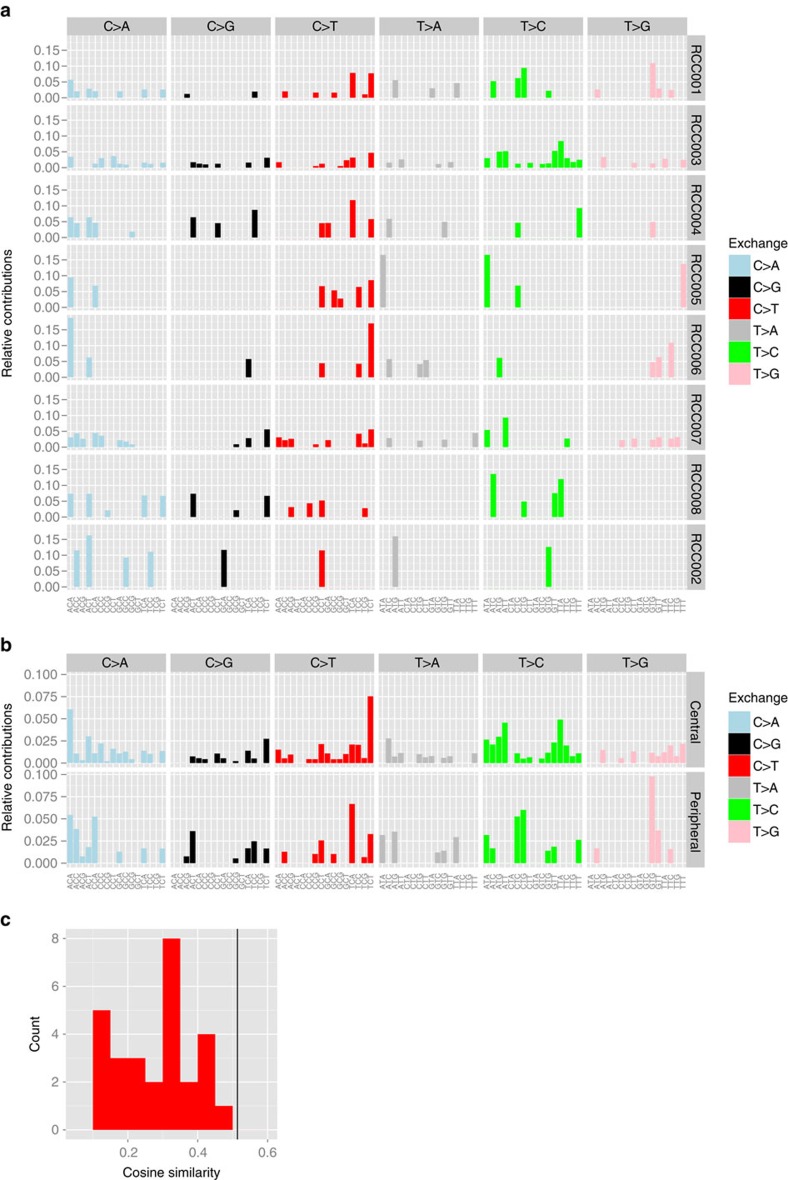
Mutational signatures do not differ between the tumour periphery and the tumour centre. (**a**,**b**) Triplet distribution of functional SNVs per patient (**a**) and per stratum (**b**), relative contributions. The *x* axis indicates the triplet context. (**c**) Cosine similarities of pair-wise comparisons of the patient-specific distributions (red histogram) and of the merged sets of central and peripheral SNVs (black vertical line).

To test whether specific mutational processes are active in either the central or the peripheral zone of the tumours, the triplet distribution of the SNVs was analysed per patient (Fig. 8a) and per stratum (Fig. 8b). Pair-wise comparisons of the patient-specific distributions revealed cosine similarities between 0.107 and 0.477, while the cosine similarity between the merged sets of central and peripheral SNVs was 0.514 (Fig. 8c), indicating higher differences in the SNV spectra between the patients than between the tumour peripheral and central areas.

Taken together, whole-exome sequencing analysis demonstrates the presence of central and peripheral zone-specific mutations, while mutational signatures do not differ spatially. Given the presence of driver mutations in both the peripheral and central areas and the absence of recurrent mutations in cell cycle or other signalling-related genes in the peripheral zone specimens, we conclude that the enhanced proliferation rate and signalling activity in the tumour peripheral zone represent mainly functional events.

## Discussion

Renal cancer is a prototypical example of a tumour entity that is characterized by extensive genomic ITH^5^,^6^. In the present report, we use quantitative image analysis and whole-exome sequencing to show that functional as well as genomic ITH can be detected at similar levels in primary ccRCCs of the lowest stage and advanced, metastatic tumours. ITH is hence not a result of malignant progression, but rather represents a feature of malignant growth *per se*. To explain these findings, we demonstrate that functional ITH follows a zonal pattern with peak proliferative and signalling activities almost exclusively in the tumour periphery. Genomic ITH was found to follow this topological pattern, since we detected various periphery- and centre-specific functional SNVs and indels in our whole-exome sequencing analysis. Functional and genomic niche formation may hence represent an early and general consequence of an uncoordinated, three-dimensional growth of a tumour within the architectural confinement of an established organ. Since such niches, that is, tumour centre and periphery, are maintained during malignant progression, the degree of ITH is comparable between small and large tumours. Our results provide strong support for the notion that genomic analyses of cancer need to take spatial differences into account.

Our whole-exome sequencing results do not contradict previous studies showing branched and converging evolution in ccRCC^5^,^6^, but they underscore that these evolutionary processes are shaped by topological niches and overlaid by functional heterogeneity. The fact that we found periphery-specific mutations could argue for a model in which tumour subclones with certain mutations that confer a selective advantage are able to populate this region (‘clone drives niche'). If the periphery-specific mutations detected do in fact confer such a growth advantage remains to be determined. However, the absence of recurrently mutated genes that would directly explain the consistently higher proliferative and signalling activity and the absence of periphery-specific mutations in three tumours suggest an important role of microenvironmental factors in niche formation and ITH (‘niche drives clone'). In this scenario, the enhanced proliferation and signalling activity in the peripheral zone would represent mainly functional events. A zonal tumour growth pattern has previously been suggested by mathematical modelling as well as three-dimensional *in vitro* co-culture experiments using breast cancer cells^14^,^15^,^16^. Since truncal drivers were predominantly found in both niches, one possible explanation for our results is that proliferation is limited in the tumour centre, for example, by spatial or other constraints, and that these constraints become more relaxed towards the tumour periphery. How this relaxation is achieved is currently unclear, but there are several possible scenarios including differences in the level of oxygen, growth factors, cytokines or interstitial fluid pressure^17^. Although we did not detect any significant differences in microvessel density in vital tumour areas of the centre and the periphery, it is still possible that local oxygen tension and/or cytokine/chemokine levels play a role, since vessel density and nutrient supply are frequently uncoupled in malignant tumours^18^. Another possibility is the ‘empty site' model that has recently been proposed and in which tumour cells proliferate preferentially when neighbouring non-tumour cells or the extracellular matrix^14^. A third possibility to explain the intratumoural niche formation shown here is co-evolution of tumour and stroma cells^19^, which would be favoured in the tumour peripheral zone. It needs to be emphasized that these mechanisms may not function independently, but may rather represent co-operating mechanisms for topological functional and genomic ITH.

Despite an enhanced proliferation in the tumour periphery, the distributions of nucleotide exchanges in their triplet contexts were more similar for the two strata than between the different patients suggesting that the mutational processes are similar in the tumour centre and periphery.

Spatial differences in ITH have been reported not only in ccRCC^5^,^6^ but also in other tumour entities such as prostate^20^,^21^,^22^,^23^, lung^24^,^25^ and breast cancer^26^,^27^. Although we did not perform multiregion sequencing and focused on relatively large single tumour regions instead, our results are in line with studies showing ITH in ccRCCs of different stage^6^. Interestingly, the application of biodiversity scores such as the Shannon or Simpson index, which are also used in the present study, has previously shown a similar extent of ITH between various breast cancers^26^,^27^. The global extent of ITH may hence not be a suitable prognostic marker, not only for RCC but also for other tumours. Nonetheless, our results encourage a more refined approach to characterize ITH under inclusion of spatial differences and intratumoural niches. In this context, it is important to point out that there is heterogeneity even within the peripheral zone, as shown by the finding that phospho-mTOR S2448 and phospho-S6RP S235/236 occupied different areas within the peripheral zone, that is, direct expression at the invasion front and expression a few cell layers away from the invasion front as shown in Fig. 6. This is remarkable since both proteins can be phosphorylated by ribosomal S6 kinase 1 (ref. 28). However, mTOR phosporylation has been implicated in a negative-feedback loop^28^, and our finding may represent a reflection of this regulatory mechanism.

Although we used HIF protein expression as surrogate for *VHL* loss, it is noteworthy that the expression of HIF proteins is also affected by other factors, for example, loss of chromosome 14q that harbours the *HIF1A* gene. HIF protein expression has been shown to be heterogeneous in the past^8^ and, furthermore, high expression of HIF-1α has been reported at the invading edge of malignant tumours similar to the results shown here^29^. HIF overexpression has been linked to the loss of the adhesion molecule E-cadherin^30^,^31^ resulting in an altered migratory behaviour, which has recently been suggested as a key factor for malignant progression^14^. Hence, potential effects of a pharmacological inhibition of migratory behaviour of tumour cells to prevent malignant progression deserve further attention. Additional translational implications of our finding would be that the use of an mTOR inhibitor could specifically target peripheral zone cells with high proliferative capacity.

In summary, our results indicate the presence of topological niches within single regions of primary ccRCCs that not only harbour region-specific mutations but are also characterized by tumour clones with unique functional properties. We provide unexpected insights into the architecture of malignant tumours and clonal evolution that have important implications for the analysis of cancer genomes in general. An integrated analysis of functional and genomic ITH appears to be required for a comprehensive analysis and better understanding of cancer genome evolution and malignant progression.

## Methods

### Tumours and immunohistochemistry

Formalin-fixed, paraffin-embedded specimens from a total of 30 primary ccRCCs (Supplementary Table 1) were retrieved from the archives of the Department of Pathology of the University of Heidelberg School of Medicine under Ethics committee vota 206/2005 and 207/2005, and informed consent by the patients. Sections were deparaffinized in xylene and rehydrated in a graded ethanol series. Antigen retrieval was performed with a steam cooker using retrieval buffer (Target Retrieval Solution, Dako). Primary antibodies used were as follows: HIF-1α (Novus Biologicals, H1alpha67, NB100-105, 1:100), HIF-2α (Novus Biologicals, NB100-122, 1:100), phospho-mTOR S2448 (Cell Signaling, 49F9, #2976, 1:100), phospho-S6RP S235/236 (Cell Signaling, #2211, 1:50), Ki-67 (Dako, MIB-1, M7240, 1:100), CD31 (Dako, JC70A, M0823, 1:100) and CD45 (Dako, 2B11+PD7/26, M0701, 1:100). Immunodetection was performed using the Histostain-Plus Detection Kit (Invitrogen) according to the manufacturer's recommendations. The immunostaining for HIF-1α has been validated in a previous study using a ccRCC with a deletion in exon 2 of the *VHL* gene^32^.

### Image quantification

Stained sections were scanned using a Nanozoomer 2.0-HT Scansystem (Hamamatsu Photonics) to generate digital whole-slide images. Quantification of the digital images was performed using the VIS software suite (Visiopharm, Hoersholm, Denmark). To facilitate the comparison of identical tumour regions stained on separate slides with different antibodies, adjacent tissue sections were aligned and virtually segmented into squares of 1 mm^2^ in size. Staining results of each square were computed using either the PNC algorithm (HIF-1α, HIF-2α and Ki-67) or the PPC algorithm (phospho-mTOR S2448 and phospho-S6RP S235/236). The latter algorithm was designed to measure every pixel per 1 mm^2^ square based on its spectral characteristics. Non-cancerous regions were excluded from quantification. Non-malignant cells within a cancerous area (for example, endothelial cells or immune cells) were not excluded under the assumption that the proportion of malignant versus non-malignant cells is comparable across tumours. Staining results for each square were expressed as either percentage positive nuclei or percentage positive pixels. If the 1 mm^2^ square had to be reduced because of non-cancerous parts, the percentage of positive pixels was normalized to the corrected area. Staining results were then depicted as heat maps.

To assess and compare heterogeneous staining, two different heterogeneity scores were used. First, the s.d. that expresses the distribution of positivity of single squares compared with average and, second, the difference between the maximum and the average positive value of squares (MAX-μ) which serves to highlight outliers within a tumour area. For the purpose of validating the used heterogeneity scores, we also applied two established biodiversity indices to our data set, that is, the Shannon and inverse Simpson index. Both indices estimate biodiversity based on the number of data categories s (=species) and the quantity of squares (=individuals) of their respective data categories. To compare the biodiversity indices between the five different biomarkers, data categories were defined dependent on the range of % PP or % PN, respectively. % PN of HIF-1α and HIF-2α range from 0 to 100% and 5% intervals were used to reach a maximum of 20 data categories per sample. % PP of phospho-mTOR S2448 and phospho-S6RP S235/236 as well as % PN of Ki-67 range roughly from 0 to 25% and therefore 2% intervals were defined to reach the same maximum of data categories (*s*=20).

The Shannon index assumes that all data categories are represented in a given sample and that they are randomly distributed:


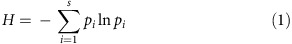


*p* is the proportion of squares of one specific data category, divided by the total number of squares found in a given sample, and *s* is the number of data categories.

The inverse Simpson index gives more weight to common data categories. Therefore, rare data categories with only a few individuals (=outliers) will not affect the diversity.


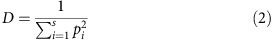


The peripheral tumour zone was defined as outermost squares of each tumour. Small lesions in which only two layers of squares were present (and hence by definition no central zone; *n*=3) were excluded from the analysis.

### Whole-exome sequencing

DNA extraction and whole-exome sequencing were performed by GATC Biotech (Konstanz, Germany) on an Illumina HiSeq 2500 platform (InView Human Exome). Fastq files were aligned against the human reference genome (build 37, version hs37d5), using bwa mem (version 0.7.8 with minimum base quality threshold set to zero [-T 0] and remaining settings left at default values), followed by coordinate sorting with bamsort (with compression option set to fast (1)) and marking duplicate read pairs with bammarkduplicates (with compression option set to best (9); both part of biobambam package version 0.0.148).

The generated bam files were used to identify SNVs by applying samtools mpileup^33^ (version 0.1.19 with parameter settings -REI -q 30 -ug) with output piped to bcftools view (version 0.1.19 with parameters settings -vgN -p 2.0). Determination of high-quality sample-specific SNVs was largely done as described in ref. 34 with additional classification into 96 categories according to their triplet of the base preceding the SNV, the actual transition/transversion and the following base. For each class, it was determined whether its SNVs exhibit a sequencing strand bias. For classes with bias, SNVs with less than two supporting reads on the strand opposite to the bias were filtered out. SNVs were called functional, when they were nonsynonymous, occurred within splice sites or lead to gain or loss of stop codons. For each category, SNV counts were determined and corrected for the triplet distribution of the human exome using the genomic coordinates of the used target capture kit (Agilent 5 without untranslated regions). Normalized counts were grouped into the two different strata (central and peripheral) and analysed both per patient to get eight patient-specific distributions and for all patients together to get two strata-specific distributions. The distributions were compared using pair-wise cosine similarity as previously described^13^.

Indels were identified using Platypus callVariants (version 0.8.1, parameter settings genIndels=1, genSNPs=1, bufferSize=1,00,000 and maxReads=1,00,00,000)^35^.

All SNVs and indels were annotated using Annovar^36^ (version as of November 2014), dbSNP (Build 141)^37^, 1000 Genomes^38^ and EVS (Exome Variant Server, NHLBI GO Exome Sequencing Project (ESP), Seattle, WA (version ESP6500SI-V2)) data.

Genome data are deposited at the European Genome-phenome Archive (EGA, http://www.ebi.ac.uk/ega/), which is hosted at the EBI, under accession number EGAS#00001001784.

### Statistical analysis

The data are summarized as median±interquartile range. The D'Agostino–Pearson omnibus test was used to determine whether the data within are consistent with a Gaussian distribution. Statistical analyses were performed with the Mann–Whitney, Kruskal–Wallis, Fisher exact test and Bernoulli trial, as appropriate. Bernoulli trial was used with probability of success of 0.51 because the number of squares in the tumour periphery versus the centre were similar (4,475 versus 4,425). Statistical significance was accepted at values of *P*<0.05. Correlation analyses were performed using Spearman correlation for non-parametric variables. Degrees of correlation were considered as followed: 0.0–0.2 (no correlation), 0.2–0.4 (weak correlation), 0.4–0.7 (moderate-to-good correlation) and 0.7–1.0 (good-to-very good correlation).

### Data availability

Genome sequencing data are deposited at the European Genome-phenome Archive (EGA, http://www.ebi.ac.uk/ega/), which is hosted at the EBI, under accession number EGAS#00001001784. All other relevant data supporting the findings of this study are either included within the article and its Supplementary Information files or available upon request from the corresponding author.

## Additional Information

**Accession codes**: The whole genome sequencing data have been deposited in the European Genome-phenome Archive (EGA, http://www.ebi.ac.uk/ega/) which is hosted at the EBI, under accession code EGAS#00001001784.

**How to cite this article:** Hoefflin, R. *et al.* Spatial niche formation but not malignant progression is a driving force for intratumoural heterogeneity. *Nat. Commun.* 7:11845 doi: 10.1038/ncomms11845 (2016).

## Supplementary Material

Supplementary Information Supplementary Figures 1-4 and Supplementary Tables 1-5.
