# Supplementary material for: Spatial niche formation but not malignant progression is a driving force for intratumoural heterogeneity
Source: Nat Commun. 2016 Jun 13;7:ncomms11845. doi: 10.1038/ncomms11845 (PMC4910022; doi:10.1038/ncomms11845)
Supplement: Supplementary Information — Supplementary Figures 1-4 and Supplementary Tables 1-5. [file ncomms11845-s1.pdf]

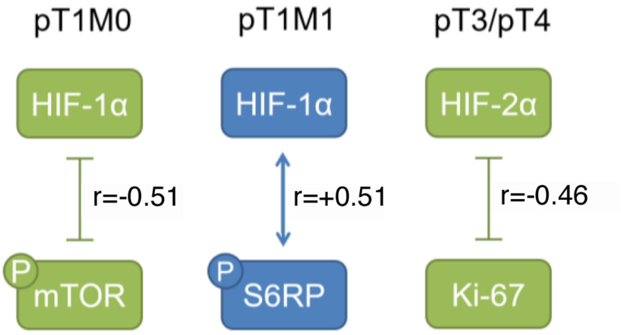

Supplementary Figure 1. Overview of changes in signaling cascades inferred from correlative marker analysis.

The correlation coefficient r demonstrates a positive (blue) or negative (green) correlation. Original data are shown in Supplementary Table 4.

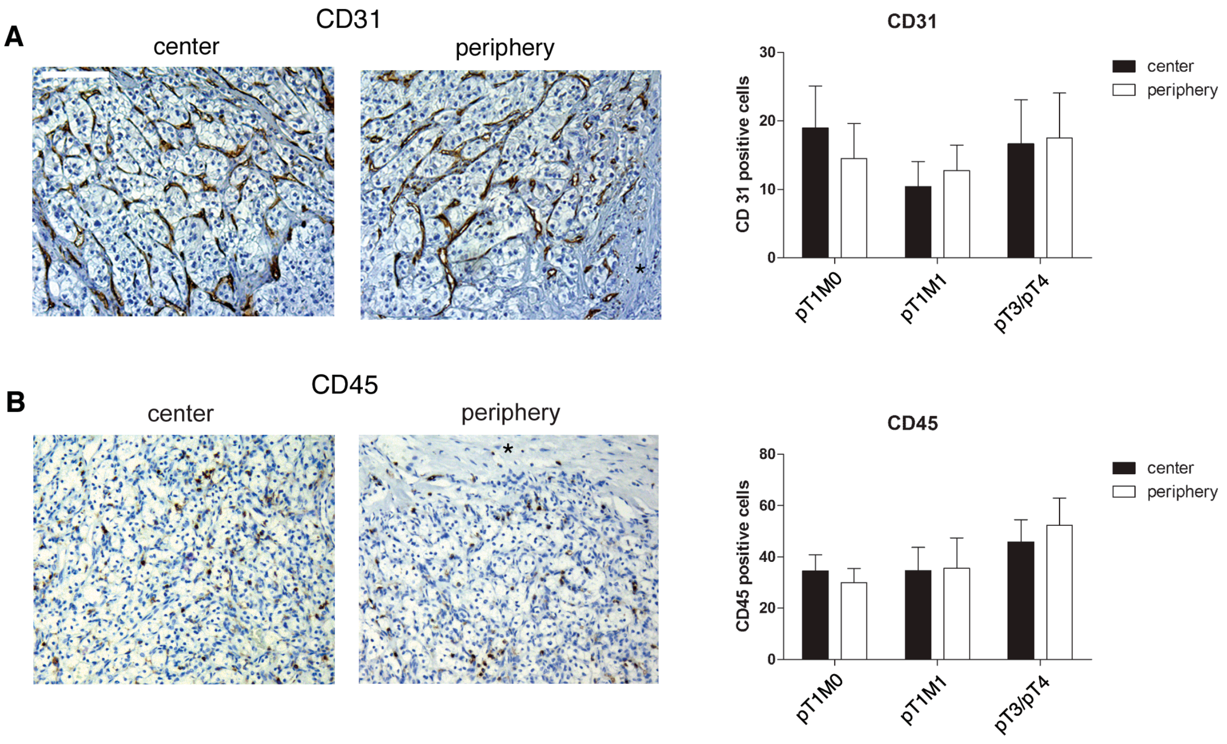

Supplementary Figure 2. Microvessel density and leukocyte admixture are similar in the tumor center and periphery.

(A,B) Representative immunohistochemical stainings for CD31 and CD45 to visualize blood vessels and leukocytes, respectively. Asterisks denote the adjacent stroma. Scale bar indicates 100 μm. Bar graphs show mean + s.e.m. of positive cells per 40x high power field (HPF) for vital (>90%) tumor areas of the center (black) and periphery (white) for tumor subgroups as indicated. At least five HPFs were counted per region and per tumor.

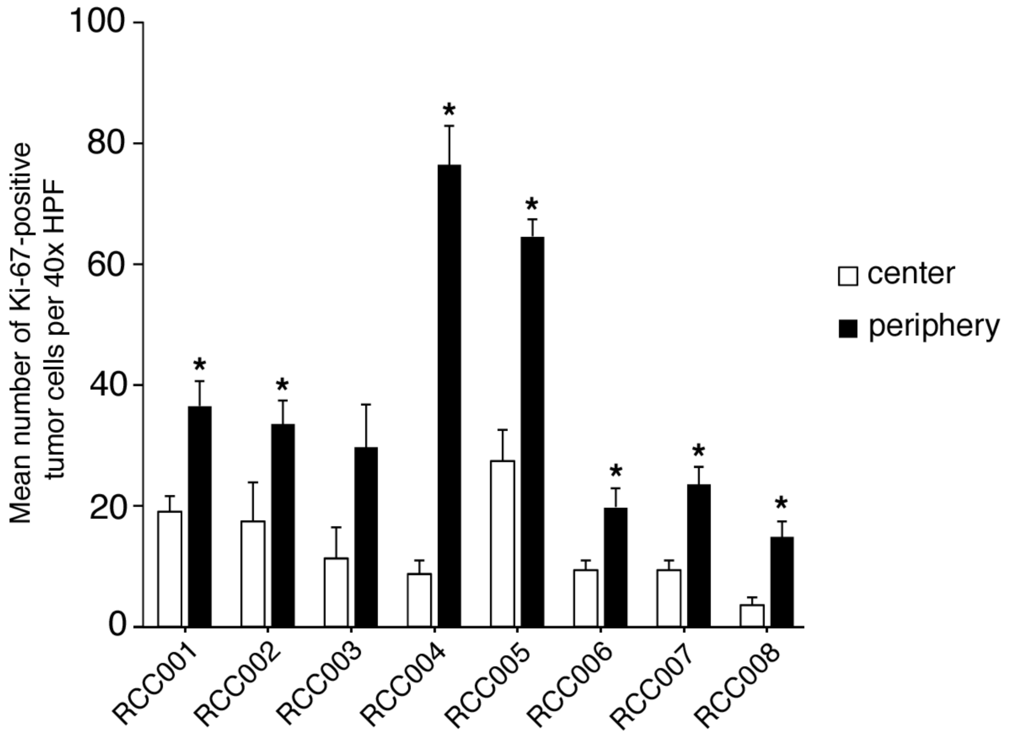

Supplementary Figure 3. Zonal pattern of functional heterogeneity in eight ccRCCs analyzed by regional whole exome sequencing.

Bar graph shows mean + s.e.m. of Ki-67-positive cells per 40x high power field (HPF) for vital (>90%) tumor areas of the center (white) and periphery (black) for the tumors indicated. At least five HPFs were counted per area and per tumor. Asterisks indicate statistically significant differences ($p<0.05$; Mann-Whitney U test).

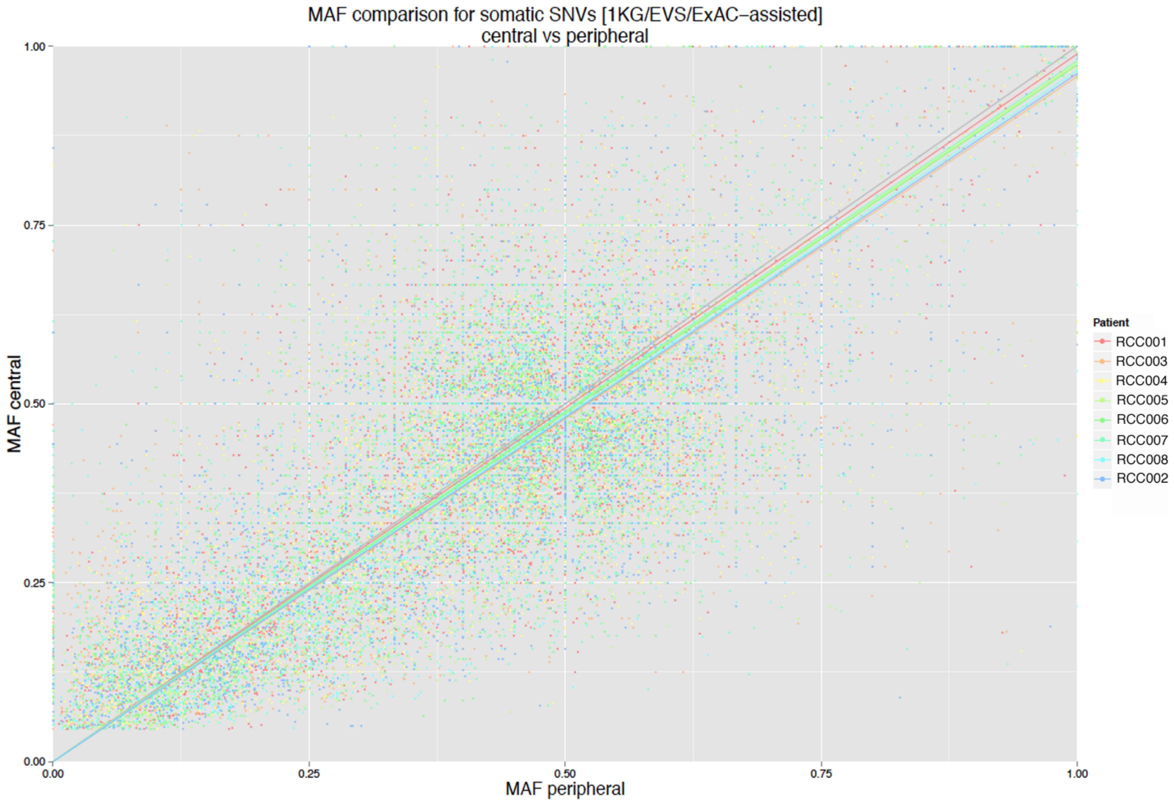

Supplementary Figure 4. Mutant allele frequencies (MAFs) in the tumor center and periphery.

Note the similar distribution of MAFs in the center and periphery indicating that results are not caused by an unequal admixture of non-tumorous cells.

Supplementary Table 1. Patient characteristics (n=30, all ccRCCs)

Parameter	n=	%
Sex		
Male	20	66.7%
Female	10	33.3%
Age at diagnosis (yrs)		
<65	20	66.7%
≥65	10	33.3%
Tumor stage		
pT1	19	63.3%
pT3/4	11	36.7%
Lymph node metastasis		
N0/pN0	28	93.3%
N1/pN1	2	6.7%
Distant metastasis		
M0	12	40%
M1	18	60%
pTNM		
pT1 N0 M0	9	30%
pT1 N0 M1	10	33.3%
pT3/4 N0 M0	3	10%
pT3/4 N0 M1	6	20%
pT3/4 N1 M1	2	6.7%
Fuhrman Grade		
G1/2	22	73.3%
G3/4	8	26.7%

Supplementary Table 2. Overview of test results

D'Agostino-Pearson Omnibus Test	pT1M0	pT1M1	pT3/pT4	all*
**negative samples**	35	30	40	105
**significant ($p \leq 0.05$)**	35	30	40	105
**non-significant ($p > 0.05$)**	0	0	0	0
**%**	78%	67%	73%	72%
**positive samples**	10	15	15	40
**significant ($p \leq 0.05$)**	0	0	0	0
**non-significant ($p > 0.05$)**	10	15	15	40
**%**	22%	33%	27%	28%

*one tumor omitted due to insufficient number of data points

Supplementary Table 3. Correlation of tumor heterogeneity with Fuhrman grade

	STDEV (median)			MAX-μ (median)		
	G1/2	G3/4	*p*-value*	G1/2	G3/4	*p*-value*
HIF-1α	10.8	6.6	0.38	24.7	15.3	0.27
HIF-2α	14.1	10.5	0.47	29.7	18.8	0.33
p-mTOR	2.2	0.3	0.08	8.8	3.5	0.21
p-S6RP	1.7	3.3	0.12	5.5	13.4	0.09
Ki-67	1.8	3.2	0.1	6.7	15.8	0.08

*Mann Whitney U test

Supplementary Table 4. Correlation between markers

pT1M0					
coefficient (r)*	p-mTOR	p-S6RP	HIF-1α	HIF-2α	Ki-67
p-mTOR		0.51	-0.51	-0.07	-0.11
p-S6RP	0.51		-0.25	-0.05	-0.38
HIF-1α	-0.51	-0.25		0.42	-0.07
HIF-2α	-0.07	-0.05	0.42		-0.27
Ki-67	-0.11	-0.38	-0.07	-0.27	
p-value	p-mTOR	p-S6RP	HIF-1α	HIF-2α	Ki-67
p-mTOR		<0.01	<0.01	0.10	0.01
p-S6RP	<0.01		<0.01	0.30	<0.01
HIF-1α	<0.01	<0.01		<0.01	0.11
HIF-2α	0.10	0.30	<0.01		<0.01
Ki-67	0.01	<0.01	0.11	<0.01	
**pT1M1**					
coefficient (r)*	p-mTOR	p-S6RP	HIF-1α	HIF-2α	Ki-67
p-mTOR		0.48	0.10	-0.10	0.24
p-S6RP	0.48		0.51	0.21	0.28
HIF-1α	0.10	0.51		0.43	0.19
HIF-2α	-0.10	0.21	0.43		-0.18
Ki-67	0.24	0.28	0.19	-0.18	
p-value	p-mTOR	p-S6 RP	HIF-1α	HIF-2α	Ki-67
p-mTOR		<0.01	<0.01	0.01	<0.01
p-S6RP	<0.01		<0.01	<0.01	<0.01
HIF-1α	<0.01	<0.01		<0.01	<0.01
HIF-2α	0,01	<0.01	<0.01		<0.01
Ki-67	<0.01	<0.01	<0.01	<0.01	
**pT3/T4**					
coefficient (r)*	p-mTOR	p-S6RP	HIF-1α	HIF-2α	Ki-67
p-mTOR		-0.09	0.35	0.16	-0.01
p-S6RP	-0.09		0.12	0.14	0.08
HIF-1α	0.35	0.12		0,24	0,11
HIF-2α	0.16	0.14	0.24		-0,46
Ki-67	-0.01	0.08	0.11	-0.46	
p-value	p-mTOR	p-S6RP	HIF-1α	HIF-2α	Ki-67
p-mTOR		0.03	<0.01	<0.01	0.84
p-S6RP	0.03		<0,01	<0.01	0.04
HIF-1α	<0.01	<0.01		<0.01	0.01
HIF-2α	<0.01	<0.01	<0.01		<0.01
Ki-67	0.84	0.04	0.01	<0.01	
**all**					
coefficient (r)*	p-mTOR	p-S6RP	HIF-1α	HIF-2α	Ki-67
p-mTOR		0.25	-0.10	-0.02	-0.01
p-S6RP	0.25		0.18	0.13	0.05
HIF-1α	-0.10	0.18		0.38	0.07
HIF-2α	-0.02	0.13	0.38		-0.29
Ki-67	-0.01	0.05	0.07	-0.29	
p-value	p-mTOR	p-S6RP	HIF-1α	HIF-2α	Ki-67
p-mTOR		<0.01	<0.01	0.52	0.76
p-S6RP	<0.01		<0.01	<0.01	0.05
HIF-1α	<0.01	<0.01		<0.01	<0.01
HIF-2α	0.52	<0.01	<0.01		<0.01
Ki-67	0.76	0.05	<0.01	<0.01	

*Spearman correlation for non-parametric variables.
Blue indicates a positive correlation, green indicates a negative correlation.

Supplementary Table 5. Patient characteristics and distribution of functional SNVs and Indels

Patient	Sex/Age	pT Stage	Grade*	Center-specific functional SNVs and Indels (n)	Periphery-specific functional SNVs (n)
RCC001	M/75	pT1a	2	4	10
RCC002	M/63	pT1a	2	3	0
RCC003	M/75	pT1a	2	24	1
RCC004	M/44	pT2a	3	7	3
RCC005	M/82	pT4	3	3	0
RCC006	F/35	pT1b	2	6	0
RCC007	F/63	pT2a	2	15	4
RCC008	F/69	pT1a	1	6	2
			total	68	20

*All RCCs with clear cell histology.
